# Kernicterus Continues to Occur in the USA Despite Concerted Preventive Efforts

**DOI:** 10.1155/crpe/5545697

**Published:** 2026-04-22

**Authors:** Bailey B. Zeiler, Timothy M. Bahr, Robin K. Ohls, Robert D. Christensen

**Affiliations:** ^1^ Department of Pediatrics, University of Utah, Salt Lake City, Utah, USA, utah.edu; ^2^ Women and Newborn Research Program, Intermountain Health, Salt Lake City, Utah, USA

## Abstract

Kernicterus spectrum disorder continues to occur in the USA, even after improvements in detecting and managing hyperbilirubinemia were brought about by the 2022 AAP revised guidelines. Three of eight new cases of kernicterus occurring in our state during the past 15 years were infants who were intentionally born at home following a low‐risk term pregnancy where the AAP guidelines were not applied. Considering the number of births per year in each setting, infants born at home are 7 times more likely to develop kernicterus than infants born in a hospital. Herein, we report our most recent case. We then provide a practical recommendation for averting future cases of kernicterus following out‐of‐hospital births. Specifically, we recommend that hospital systems and/or health departments supply all midwives performing out‐of‐hospital births with the equipment and training necessary to comply with the AAP guidelines by quantitatively assessing hyperbilirubinemia on every neonate 24–48 h following birth. We propose that this noninvasive, simple, and rapid assessment should be free of charge to the midwife and to the family. We maintain that if this is done routinely after every out‐of‐hospital birth, it will identify the neonates who need phototherapy and who sometimes also need other medical care to prevent extreme hyperbilirubinemia. We believe that such a program will prevent acute bilirubin encephalopathy and kernicterus and that without such a program, the dreadful condition of kernicterus spectrum disorder will continue to occur among our population of infants born at home.

## 1. Introduction

Extreme neonatal hyperbilirubinemia can cause acute bilirubin encephalopathy, which can evolve into a permanent form of brain damage known as the kernicterus spectrum disorder [1, 2]. There is no cure for kernicterus; therefore, tremendous efforts have been made toward its prevention [3, 4], with the intent that it might become a “never event” [5]. Landmark publications aimed at preventing kernicterus include the 2004 American Academy of Pediatrics (AAP) guidelines for hyperbilirubinemia management [3] and the improvements made in the 2022 AAP revised guidelines [4]. The goal of both sets of guidelines was to prevent kernicterus [3, 4].

In 2018, we reported seven new cases of kernicterus occurring in Utah between 2009 and 2018 [6]. Two were intentional home births. All seven had hemolytic disorders, yet none had a family history of a hemolytic condition that would have informed caregivers of their potential risk for extreme hyperbilirubinemia after a home birth. One of the two home births was found to have pyruvate kinase deficiency (Homozygous 1529A [R510Gln] mutations). The other had hereditary spherocytosis (compound heterozygous mutations in Band 3 [E40K] and beta spectrin [S1763G]) [6].

In 2022, the National Center for Health Statistics reported that home births in the USA were increasing and had reached their highest level in 30 years; 1.26% or 51,000 per year [7]. In the state of Utah, the percentage of home births is higher than the national average, at 4.4% of all births, or 1700–1900 annually [8]. For a variety of reasons, some families desire a home birth [9, 10]. However, out‐of‐hospital births can present a challenge that is not always met by the AAP hyperbilirubinemia guidelines, simply because they are not applied. A 2020 AAP Committee on Fetus and Newborn policy statement reads, “All newborn infants [including those born at home] should be assessed for risk of hyperbilirubinemia and undergo bilirubin screening between 24 and 48 h after birth. The bilirubin value should be plotted on the hour‐specific nomogram to determine the risk of severe hyperbilirubinemia and the need for repeat determinations” [10]. However, we are not currently providing the midwives in our state with the training and equipment they need to comply with the AAP hyperbilirubinemia guidelines [4]. We know this because we recently had another case of kernicterus spectrum disorder after a home birth in Utah. We report the case herein, along with a recommendation that we believe would eliminate this catastrophe among future out‐of‐hospital births.

## 2. Case Presentation

A male was born at home at 36 weeks 3 days gestation to a 33‐year‐old, Gravida 5, Para 3‐0‐1‐3, blood type O (+), GBS‐negative mother who received prenatal care. Birth weight was 2.7 kg (6 lb 0 oz). He was evaluated by the midwife after delivery and said to be normal. Because the mother’s milk was minimal for the first few days, he was fed donor breastmilk obtained from a neighbor, receiving 1–1.5 oz every 2–3 h by bottle. His parents reported good urine and stool output. He was not evaluated again until Day 5.

On Day 5, the mother noted one episode of him staring upward for 15–20 s and called her midwife. The midwife noted that he was jaundiced and advised her to go to the emergency department. There, his weight was 2.6 kg (5 lb. 12 oz), and total serum bilirubin was 37.2 mg/dL with a direct of 2.3 mg/dL. Hemoglobin was 18.6 g/dL, and hematocrit was 53.6%. Blood type was O (+), and DAT was positive. An IV was placed, and 10 mL/kg normal saline was given by bolus. The emergency physician contacted the closest NICU, which stated that it would be unable to perform an exchange transfusion; consequently, the infant was transported by air to a Level IV NICU. Prior to transport, a second saline bolus was given, D10W was infused at 100 mL/kg/day, and phototherapy was begun and continued while en route and on subsequent NICU admission.

Upon arrival to the referred hospital, he appeared irritable and was hypertonic with decerebrate posturing. He intermittently cried but was consoled with a pacifier. He received 1 g/kg IVIG, and ampicillin and gentamicin were begun after drawing a blood culture. Head ultrasound showed no intracranial hemorrhage. Three hours after initial labs, repeat bilirubin was 31.7 mg/dL. His PT was 23.4 s, PTT 56 s, fibrinogen 327 mg/dL, so 20 mL/kg FFP was given. Anti‐E antibodies were coating his red blood cells, and blood type O, Rh negative, antigen e/e blood was used for the exchange transfusion, beginning 10 h after initial presentation to the ED. Prior to starting the exchange transfusion, bilirubin was 25.1 mg/dL. A total of 420 mL (160 mL/kg) of blood was removed, with an equal amount of donor blood given in 5 mL aliquots over a period of 5 h. After the exchange transfusion, bilirubin was 19.5 mg/dL, and the EEG was normal.

Three hours later, his bilirubin was 13.2 mg/dL. This rose to 14.1 mg/dL 7 h later, despite quadruple phototherapy. A second dose of IVIG was given. His bilirubin level then decreased to 10.6 mg/dL and continued to decline thereafter. His neurologic examination improved. The blood culture was positive after 24 h for GBS and MSSA. Lumbar puncture was bloody, and the ampicillin was increased to meningitic dosing. Four days later, a brain MRI with and without contrast showed “bilateral symmetric signal abnormality within the globus pallidus, subthalamic nuclei, and central midbrain, consistent with acute bilirubin encephalopathy and possible additional involvement of the hippocampal formations. No MR features of meningitis were identified.” He completed a 14‐day course of antibiotics. He was subsequently diagnosed by the audiology department as having auditory neuropathy spectrum disorder. At 2 months, he had difficulty with visual attention/focus and was diagnosed with developmental delay and hearing loss.

## 3. Discussion

The 2022 revised AAP hyperbilirubinemia guidelines streamlined the management of hyperbilirubinemia and led to fewer interventions [4]. For example, Balasubramanian et al. reported that the benefits of adopting the new guidelines in their hospital included a 64% reduction in serum bilirubin draws, a 51% decrease in phototherapy sessions, and a 35% reduction in readmissions for phototherapy [11]. However, reporting from the Pediatric Health Information System database (44 children’s hospitals across the USA), Jameel et al. found more new kernicterus cases (ICD 10 Codes P57.9 and P57.8) occurring during the year following the introduction of the 2022 guidelines (eight cases) than in any of the four previous years (range 1–6 new cases per year, *p* = 0.138) [12]. This trend, although not statistically significant, should be tracked. If the incidence of kernicterus is not falling, as all hope it will, or is indeed rising, perhaps one reason is that some cases are occurring in populations where the AAP guidelines are not being applied, such as out‐of‐hospital deliveries.

The AAP states that midwives attending home births should be American Midwifery Certification Board (AMCB)–certified [13]. We suggest that as part of the certification process, midwives should be instructed to conduct at least one home visit during the 24–48 h following the birth, where they make one of the following three noninvasive, rapid measurements to assess bilirubin and/or detect a hemolytic condition (Figure 1). Moreover, we suggest that it is the responsibility of society to provide midwives with the equipment needed and the training to be compliant with the AAP directives for hyperbilirubinemia detection [4, 10].

FIGURE 1Three means whereby a midwife, visiting the home 24–48 h after a home birth, can recognize and quantify pathological jaundice and/or hemolysis and facilitate treatment, thereby preventing extreme hyperbilirubinemia and kernicterus spectrum disorder. (Original artwork by Jeni Walker, Division of Neonatology, University of Utah, no copyright). (a) Noninvasive estimation of serum bilirubin using a transcutaneous bilirubinometer. (b) Noninvasive estimation of serum bilirubin using a smartphone device. (c) Noninvasive quantification of the hemolytic rate by ETCOc.

**Figure:**
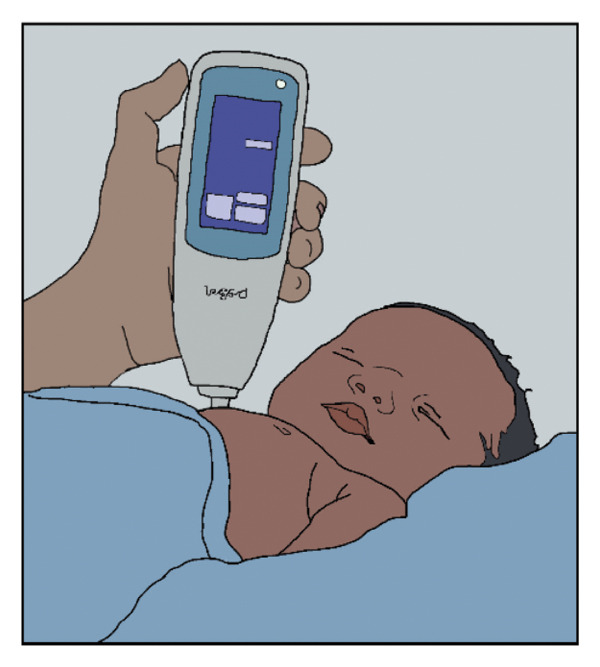
(a)

**Figure:**
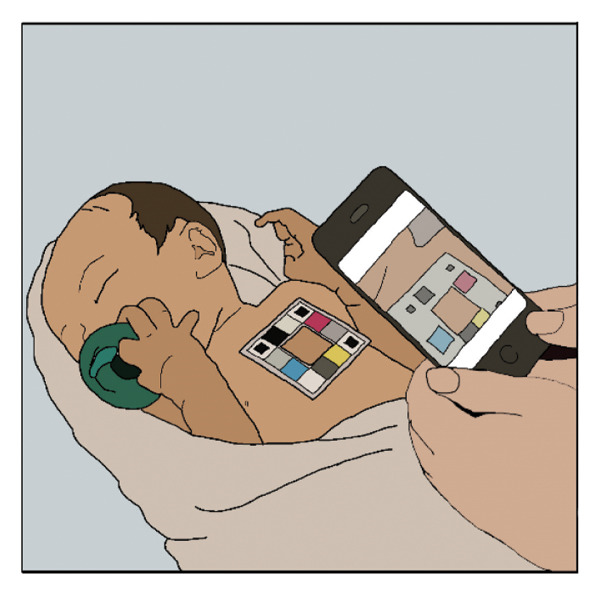
(b)

**Figure:**
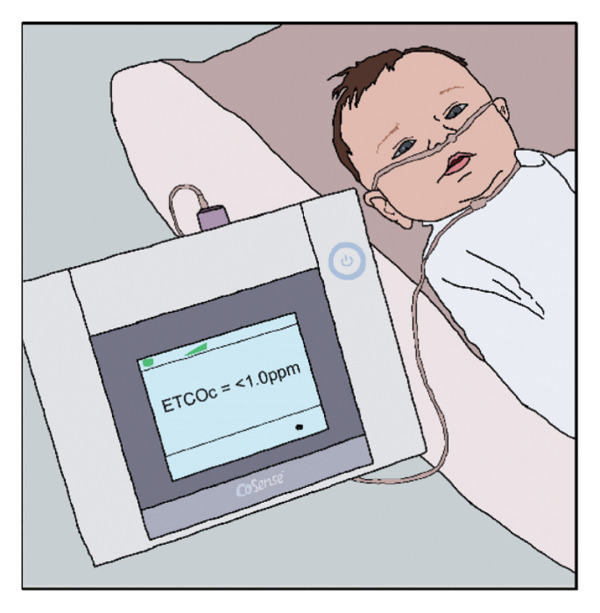
(c)

Identifying hyperbilirubinemia and/or significant hemolysis 24–48 h after home birth would foster appropriate phototherapy, and sometimes additional medical care, to avoid extreme hyperbilirubinemia, bilirubin encephalopathy, and the kernicterus spectrum disorder. We propose that midwives be provided with one of the following three methods to accomplish this.

1. Transcutaneous bilirubin measurement. Transcutaneous bilirubin measurements have the advantage of not requiring phlebotomy or a clinical laboratory. Measurements can be made in the home with a rapid, simple, noninvasive result. This technology has been extensively studied and validated [14, 15]. The midwife could make the measurement and compare the bilirubin value obtained with the guidelines of the 2022 AAP report [4] to determine whether phototherapy is needed, and if so, facilitate that care from a medical provider.
2. Smartphone estimate of total serum bilirubin level. Midwives in the European Union, many parts of Asia, and Mexico have access to a noninvasive, inexpensive, and accurate method of estimating bilirubin level using a smartphone app called Picterus [16]. While not yet FDA‐approved for use in the USA, Picterus can rapidly assess the bilirubin level in the home setting, and that value can be compared with the guidelines of the 2022 AAP report [4] to determine whether phototherapy is needed.
3. Quantification of the hemolytic rate using end‐tidal CO. In our series, and in our practice, all cases of kernicterus are the result of *hemolytic* jaundice [6], with the exceedingly rare condition Crigler–Najjar syndrome Type 1 being an exception [17]. A midwife visiting the home 24–48 h after birth could measure the hemolytic rate noninvasively, rapidly, and economically using a nasal cannula, a process taking one to two minutes. Values ≤ 1.7 ppm are normal for newborn infants; thus, a measurement above 1.7 ppm indicates hemolysis. Values ≥ 2.5 ppm should come to the attention of a pediatric provider because these indicate significant hemolysis [18–20].

During a home visit by a midwife or another caregiver, 24–48 h after birth, one of the three rapid noninvasive measurements could be made, with any abnormal or questionable result reported to a medical provider. If this were routinely practiced in our state, it is unlikely that the two infants in our previous report [6] and the one in our present report would have the lifetime handicapping conditions they now do.

Challenges to widespread implementation include various certification sources for midwives, costs of equipment and training, and access to devices. Transcutaneous bilirubinometers could be widely available across different healthcare systems; however, they are the most expensive option. End‐tidal CO monitors could also be widely available at an intermediate cost. Finally, Picterus [16] is widely available in other countries, even in low‐resource settings, and is inexpensive. However, this option is not currently available in the USA.

A dictum displayed on some online law office postings is, “Kernicterus indicates medical malpractice.” Indeed, kernicterus compensation claims are among the highest and most successful jury awards in pediatrics [21]. However, much more important than the effect that lowering kernicterus rates would have on reducing malpractice claims is the effect it would have on reducing the number of individuals stricken with lifelong handicapping conditions because of what we all wish would become a “never event.”

## Funding

No funding was received for this manuscript.

## Disclosure

This case has been orally presented at the Western Society for Pediatric Research in Carmel, CA, on January 17, 2026.

## Consent

No written consent has been obtained from the patients as there is no patient identifiable data included in this case report.

## Conflicts of Interest

The authors declare no conflicts of interest.

## Data Availability

The data that support the findings of this study are available from the corresponding author upon reasonable request.
